# Selective bacteriophages reduce the emergence of resistant bacteria in bacteriophage-antibiotic combination therapy

**DOI:** 10.1128/spectrum.00427-23

**Published:** 2024-05-02

**Authors:** Aa Haeruman Azam, Koji Sato, Kazuhiko Miyanaga, Tomohiro Nakamura, Shinjiro Ojima, Kohei Kondo, Azumi Tamura, Wakana Yamashita, Yasunori Tanji, Kotaro Kiga

**Affiliations:** 1 Therapeutic Drugs and Vaccine Development Research Center, National Institute of Infectious Diseases, Toyama-ku, Shinjuku, Tokyo, Japan; 2 School of Life Science and Technology, Tokyo Institute of Technology, Nagatsutacho, Yokohama, Japan; 3 Division of Bacteriology, Department of Infection and Immunity, Jichi Medical University, Shimotsukeshi, Tochigi, Japan; The University of Tennessee Knoxville, Knoxville, Tennessee, USA

**Keywords:** bacteriophage therapy, O157:H7, antimicrobials, fosfomycin, drug resistance evolution, drug resistance mechanisms, outer membrane proteins, gut microbiota, diarrhea

## Abstract

*Escherichia coli* O157:H7 is a globally important foodborne pathogen with implications for food safety. Antibiotic treatment for O157 may potentially contribute to the exacerbation of hemolytic uremic syndrome, and the increasing prevalence of antibiotic-resistant strains necessitates the development of new treatment strategies. In this study, the bactericidal effects and resistance development of antibiotic and bacteriophage monotherapy were compared with those of combination therapy against O157. Experiments involving continuous exposure of O157 to phages and antibiotics, along with genetic deletion studies, revealed that the deletion of *glpT* and *uhpT* significantly increased resistance to fosfomycin. Furthermore, we found that OmpC functions as a receptor for the PP01 phage, which infects O157, and FhuA functions as a receptor for the newly isolated SP15 phage, targeting O157. In the *glpT* and *uhpT* deletion mutants, additional deletion in *ompC*, the receptor for the PP01 phage, increased resistance to fosfomycin. These findings suggest that specific phages may contribute to antibiotic resistance by selecting the emergence of gene mutations responsible for both phage and antibiotic resistance. While combination therapy with phages and antibiotics holds promise for the treatment of bacterial infections, careful consideration of phage selection is necessary.

**IMPORTANCE:**

The combination treatment of fosfomycin and bacteriophages against *Escherichia coli* O157 demonstrated superior bactericidal efficacy compared to monotherapy, effectively suppressing the emergence of resistance. However, mutations selected by phage PP01 led to enhanced resistance not only to the phage but also to fosfomycin. These findings underscore the importance of exercising caution in selecting phages for combination therapy, as resistance selected by specific phages may increase the risk of developing antibiotic resistance.

## INTRODUCTION

Antibiotics have been used in clinical practice to fight infectious diseases since their discovery approximately a century ago (1). However, the extensive use of antibiotics and the lack of effective control treatments have rapidly increased the emergence of antimicrobial-resistant (AMR) bacteria. It is projected that by 2050, the number of deaths caused by AMR infections will outnumber the deaths caused by cancer if alternative treatments are unavailable (2). Phage therapy involves the use of bacteriophages—viruses that specifically infect bacteria—and it has attracted significant attention as a possible alternative to combat the AMR problem (3–11). However, the emergence of phage-resistant bacteria has been reported in several studies, including clinical experiments in humans (12–14). Therefore, rather than replacing antibiotics with phages, combination therapy is being used to combat AMR. Combination therapy is known to be less likely to produce resistance than either therapy alone.

Enterohemorrhagic *Escherichia coli* serogroup O157:H7 is a worldwide source of infection that causes bloody diarrhea and hemolytic uremic syndrome (HUS) in humans and animals (15–17). Most *E. coli* O157 infections in humans are foodborne diseases transmitted through domestic animals as reservoirs of O157 (17, 18). AMR in O157 is notably prevalent, particularly in developing countries (19–21). Using phages to control pathogenic organisms in the gastrointestinal tract is a promising strategy, and several studies have been successful in animal models (22–25).

Fosfomycin, an antibiotic produced by *Streptomyces* sp. in 1969, has been used for many years (26). As the number and types of drug-resistant bacteria increase, fosfomycin is currently attracting attention as an effective antibacterial drug against multidrug-resistant bacteria, owing to its low molecular weight (molecular weight: 138) compared to other antibiotics (27, 28). Currently, fosfomycin is used as a standard treatment for urinary tract infections caused by *E. coli* and fecal streptococci and for HUS caused by enterohemorrhagic *E. coli* (29, 30). Studies have also reported the efficacy of fosfomycin against multidrug-resistant *E. coli* and *Pseudomonas aeruginosa* (31, 32). However, fosfomycin alone may increase the probability of developing HUS and should be used cautiously in the clinical setting.

For both antibiotics and phages, tradeoffs occur with the acquisition of resistance. While changes in the membrane structure and reduced growth rates are common outcomes in both cases, the mechanisms underlying phage and antibiotic resistance are typically different. This implies that administering a combination of antibiotics and phages simultaneously may be more effective than individual administration because distinct tradeoffs are required for the bacteria in each case. Although the simultaneous administration of antibiotics and phages may be effective against O157, it is anticipated that O157 strains resistant to both phages and antibiotics will arise.

Therefore, our aim was to evaluate the potential utility of two different phages, PP01 and SP15, which infect O157, and analyze the underlying resistance mechanisms of bacteria exposed to the combination of these phages and fosfomycin, as well as to each antimicrobial individually.

## MATERIALS AND METHODS

### Media and buffers

All experiments were conducted using Mueller-Hinton broth (2 g beef extract, 17.5 g acid digest casein, and 1.5 g soluble starch per liter) and Luria Bertani (LB) broth (10 g polypeptone, 10 g sodium chloride, and 5 g yeast extract per liter). In accordance with the method used for testing drug susceptibility to fosfomycin, glucose-6-phosphate (G6P) was added after autoclaving the medium to achieve a final concentration of 25 µg/mL (33). Hereafter, this medium will be referred to as MHB-G6P. Fosfomycin was dissolved in sterile water, sterilized through a 0.22-µm filter, and stored at −20°C. Phosphate-buffered saline (PBS) (8.0 g NaCl, 0.2 g KCl, 1.44 g Na_2_HPO_4_, and 0.24 g KH_2_PO_4_) was used for dilution of the bacteria solution, and sodium-magnesium (SM) buffer [5.8 g NaCl, 0.2 g MgSO_4_.7H_2_O, 50 mL 1 M Tris-HCl (pH. 7.5), and 5 mL of 2% (wt/vol) gelatin] was used for dilution of the phage solution.

### Isolation of the phages

Phage SP15 was isolated from sewage influent obtained from a municipal wastewater treatment plant in Tokyo using *E. coli* O157:H7 as the propagation host, using the double-layer agar-plating method. Phage PP01 was obtained and comprehensively studied in our prior study (34, 35). The phages were propagated and purified using a previously described method (36). Briefly, the purified phage was propagated by mixing 1% of the overnight culture of O157 in liquid LB and incubated overnight at 37°C. Host cells were removed through centrifugation (11,000 × *g*, 20 min, 4°C) before phage concentration using the polyethylene glycol 6000-NaCl (PEG-NaCl) method and filtered through a 0.22-µm Millex-GP filter (Merck, Millipore, Darmstadt, Germany).

### Bacteria and phages

The strains and phages used in these experiments are listed in Table S1. *E. coli* O157:H7 ATCC 43888 (hereafter referred to as O157) has the same serotype as pathogenic *E. coli* O157:H7 but is nonpathogenic as it does not possess the Shiga toxin genes, *stx1* and *stx2* (37). PP01 and SP15 are lytic phages that can infect several O157 strains; hence, the phages were propagated using O157 as a host. Phage solutions were prepared using the overlaid agar plate method, as previously described (38–40). Briefly, 100 µL each of a 10^5^–10^6^ plaque-forming units (PFU)/mL phage solution and O157 pre-culture solution was mixed. The mixture was then added to 3 mL of top agar (LB, 0.5% agar, and 1 mM CaCl_2_) dissolved at 45°C, layered onto LB plates, and incubated at 37°C overnight. Next, 4 mL of SM buffer was added to the plate on which the plaque was formed, and only the upper layer was scraped off. The supernatant was then collected after centrifugation (10,000 × *g*, 5 min, 4°C). Finally, chloroform was added to a final concentration of 2% (vol/vol), after which the phage solution was stored at 4°C. Phage concentration was measured using the plaque assay method. Diluted phage solution (10^3^–10^4^ PFU/mL) and O157 pre-culture solution were mixed in 100 µL portions, added to 3 mL of top agar dissolved at 45°C, layered on an LB plate, and incubated at 37°C overnight. The phage solution concentration was determined by counting the number of plaques.

### Isolation of resistant strains

O157 was passaged in a medium containing fosfomycin and phage (PP01 or SP15) to screen for antibiotic- and phage-resistant strains. L-shaped test tubes containing 4 mL of MHB-G6P were inoculated with 10^7^ colony-forming units (CFU)/mL of O157 overnight culture, and fosfomycin (4 µg/mL) and PP01 or SP15 (10^7^ PFU/mL) were added alone or in combination 1 h after bacterial addition. Those to which neither fosfomycin nor phage was added were used as controls. Cultures were incubated using a small shaking culture device (TVS062CA BioPhoto recorder, ADVANTEC, Tokyo, Japan) at 37°C with shaking at 40 rpm, and the turbidity (OD_660_) was measured every 15 min. The incubation continued until the bacterial growth reached a stationary phase, which was at 48 h when used alone and at 72 h when used in combination. After incubation, the phage concentration in the culture medium was measured using the plaque assay method for samples with added phages. Subsequently, for the control and phage-treated groups from the previous round, a 1% inoculation into 4 mL of new MHB-G6P was carried out. The cultures were then incubated under the same conditions for the next round. For samples to which fosfomycin was added, the minimum inhibitory concentration (MIC) was measured on the resistant clone isolated at the end of each round. Subsequently, fosfomycin was introduced into 4 mL of fresh MHB-G6P to achieve the same concentration as the observed MIC of the bacteria isolated from the previous round. This prepared medium was then utilized for the subsequent round of culture. This procedure was repeated for five rounds. The culture was centrifuged (10,000 × *g*, 5 min, 4°C), and the resulting supernatant was stored as phage stock, while the pellet was stored as bacterial stock in 15% glycerol at 4°C and −60°C, respectively. To isolate the resistant clone, isolation was performed for each stock obtained from the passage co-culture. A portion of each glycerol stock was streaked on an MHB-G6P plate, incubated overnight at 37°C. Afterward, one single colony was taken, inoculated into 2 mL of MHB-G6P, and incubated overnight at 37°C, 120 rpm. The same experiment was performed three times (three runs) with phage-only addition (PP01, SP15) and five times (five runs) with fosfomycin addition (fosfomycin alone, PP01 + fosfomycin, and SP15 + fosfomycin).

### Transmission electron microscopy imaging of phages

Phages were observed using transmission electron microscopy (TEM), as described previously (41). Briefly, the phage solution was concentrated using PEG-NaCl precipitation (10% PEG 6000, 0.1 M NaCl). The PEG-NaCl concentrated phage lysate was further purified through cesium chloride (CsCl) step centrifugation (step densities: 1.46, 1.55, and 1.63 g/mL). Then, the concentrated phage suspension (10^9^ PFU/mL) was spotted onto a hydrophilic plastic carbon-coated copper grid (Nissin EM Corporation, Tokyo, Japan). Phages were allowed to adsorb for 1 min before removing the excess samples. Subsequently, 10 mL of distilled water was spotted onto the grid and removed quickly. Phages were stained with 2% uranyl acetate or an EM Stainer (Nissin EM Corporation). Excess stain was removed after 1 min, and the grid was allowed to air dry for 30 min before observation using a JEOL JEM-1400Plus (JEOL, Tokyo, Japan) operating at 80 kV.

### Characterization of phage growth and determination of phage host range

**Fig 1:**
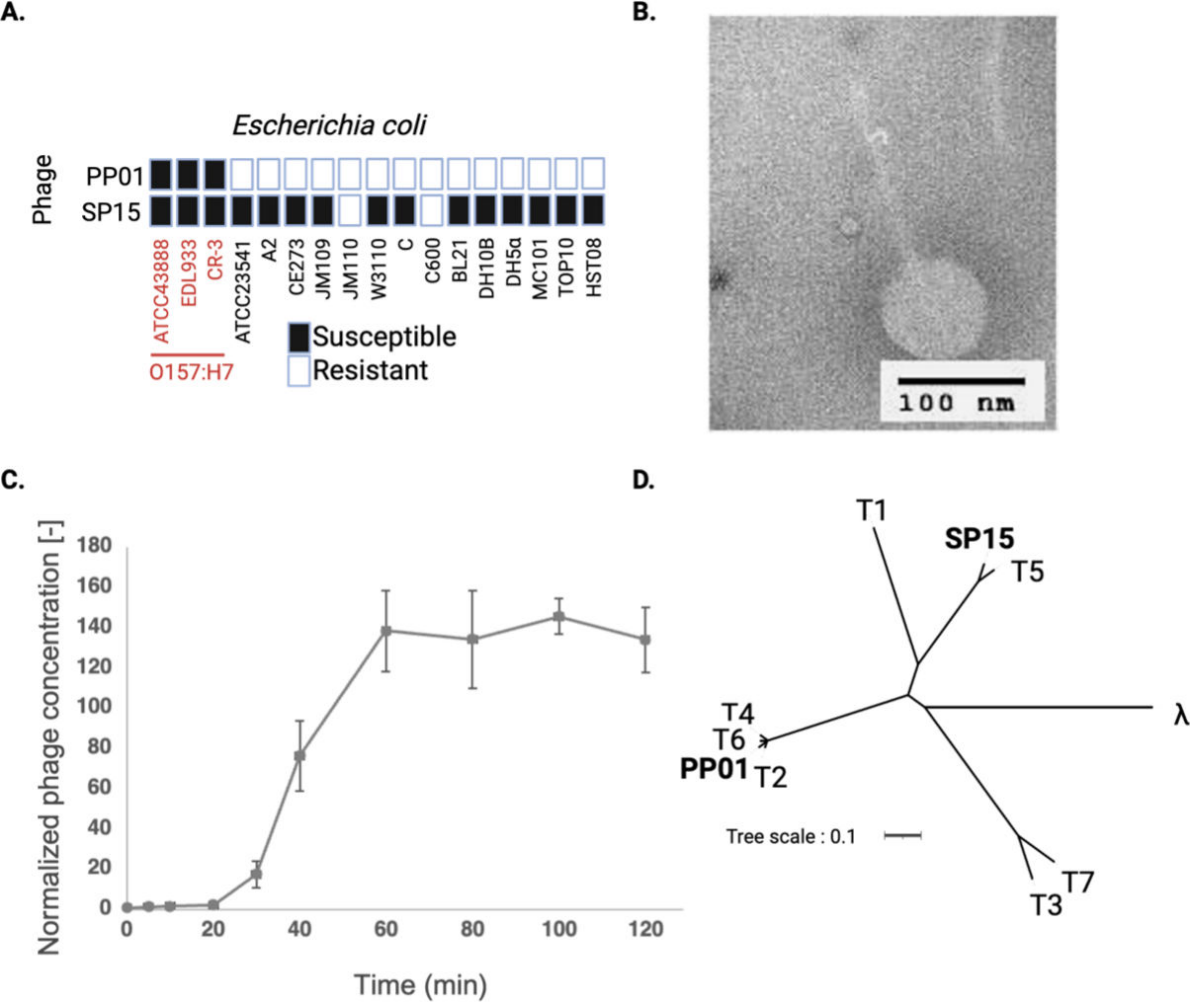
Characterization of phage SP15. (**A**) Host range of SP15 phage against various *Escherichia coli* strains. Bacterial strains in red and black letters indicate O157 and non-O157 strains, respectively. The previously isolated phage PP01 was used for comparison. (**B**) Morphological observation of SP15 under TEM. (**C**) One-step growth of SP15. (**D**) Phylogenetic tree of PP01 and SP15 among T-series and lambda phages.

A one-step growth curve was constructed to determine the burst size and latent period, as previously described, with some modifications (41). Briefly, the phages were added to a refreshed overnight culture of bacteria (OD_660_ = 1) at a multiplicity of infection (MOI) of 0.01 and incubated at 37°C for 10 min, with shaking at 120 rpm. The unbound phages were removed through centrifugation and washed five times with chilled LB medium. Phage-infected cells were incubated at 37°C for 160 min. The enumeration of phages at 0, 5, 10, 20, 30, 40, 60, 80, 100, 120, 140, and 160 min of incubation was conducted using the double-layer agar method. The host range was determined using 17 strains of *E. coli* (Fig. 1A) through a spot test wherein 2.5 µL of 10^7^ PFU/mL phage was dropped on a bacterial lawn.

### Evaluation of phage infectivity through spot testing

To evaluate the infectivity of the phages, a spot test was performed. Specifically, 100 µL of the overnight culture of O157 (wild-type or resistant clone) was added to 3 mL of LB top agar, poured onto LB plates, and allowed to dry for 10 min. Then, 5 µL of 10^9^ PFU/mL phage (wild-type or mutant phage) was dropped onto the plate and allowed to stand until it dried. After overnight incubation at 37°C, the formation of inhibition zones was recorded.

### Phage adsorption assay

The adsorption efficiency of phages on O157 was measured by titrating the free phages in the supernatant after 20 min of cell-phage contact at an MOI of 0.01. Then, 100 µL of the cell-phage solution was sampled and immediately added to 9.9 mL of chilled SM buffer. The solution was gently vortexed before extracting 1 mL for centrifugation (10,000 × *g*, 5 min, 4°C) to remove the bacterial cells before titrating the phage concentration. Adsorption efficiency was calculated by dividing the number of adsorbed phages by the initial number. Statistical analysis was performed using a two-tailed Student’s *t*-test using Microsoft Excel.

### MIC measurement test

To determine the susceptibility of O157 to fosfomycin, an MIC assay test was performed. The measurements were performed according to the method prescribed by the Clinical and Laboratory Standards Institute (33). Briefly, the O157 culture medium was diluted in PBS to 10^8^ CFU/mL. Subsequently, MHB-G6P plates containing fosfomycin at twofold diluted concentrations (0, 1, 2, 4, … µg/mL) were prepared, and 1 µL of the diluted culture medium was dropped onto the plates. After drying, the plates were incubated at 35°C for 16–20 h, and the MIC was defined as the minimum concentration of fosfomycin at which no colonies grew.

### Determination of the specific growth rate of resistant strains

The specific growth rate in the absence of antibiotics and phages was determined to clarify the fitness cost of the resistant strains obtained under each condition. L-shaped test tubes containing 4 mL of LB were inoculated with wild-type O157 or the resistant strain at 10^7^ CFU/mL and incubated at 37°C and 40 rpm for 12 h. Turbidity (OD_660_) was measured every 15 min during incubation, and the specific growth rate was determined when the OD_660_ value ranged from 0.5 to 1.1, which was considered the growth log phase.

### Genome extraction and whole-genome analysis of bacteria and phages

The GenElute Bacterial Genomic DNA Purification Kit (Sigma-Aldrich, USA) was used for bacterial genome extraction, and the phage DNA Extraction Kit (Norgen Biotex, Canada) was used for phage genome extraction, according to the manufacturer’s protocol. Whole-genome sequencing, encompassing library preparation, was conducted utilizing the Whole-Genome Analysis Service offered by BGI Japan, Inc., employing the Illumina Hiseq 2500 platform. The whole-genome sequence data of wild-type O157 (ATCC 43888) are available at the National Center for Biotechnology Information (NCBI) (Accession number: CP041623). The genome sequence data were compared to those of the same strain in our laboratory. Therefore, we assembled the sequence data of the wild-type and resistant mutant strains held in our laboratory using BWA (ver. 0.7.17), Samtools (ver. 0.1.19), and Pilon (ver. 1.23), with strains registered in the NCBI database as references (42). Based on this assembled wild-type strain data, mapping was performed using the above software to identify mutations in the resistant strains. The genome data of PP01 and SP15 can be accessed at NCBI using the accession numbers LC348379 and AP019559, respectively.

### Genetic manipulation in O157

The primers used in this study are listed in Table S2. To delete each gene (*uhpT, glpT, ompC,* and *fhuA*) from wild-type O157, recombinant plasmids were constructed using the primers listed in Table S2. Recombination templates were prepared using overlap extension PCR to generate the deletion template.

Plasmid pKOV (Addgene, USA) was used to delete the gene of interest following the established protocol (43). Plasmids and recombination templates were treated with the restriction enzymes BamHI and SalI (New England Biolabs, USA) and ligated with T4 ligase (New England Biolabs, USA). The ligation product was introduced into *E. coli* JM109 using the heat shock method and cultured on a chloramphenicol LB plate. Homologous recombination of the target genes in the O157 genome using pKOV was performed following an established protocol, with some modifications (43). Using electroporation (1.8 kV, 25 µF, and 200 Ω), the plasmid was electroporated into O157 cells, plated on a chloramphenicol LB plate, and incubated at 30°C overnight. One to three obtained colonies were suspended in 1 mL PBS, streaked on chloramphenicol LB plates, and incubated at 43°C overnight for plasmid integration. To increase transfection efficiency, incubation at 43°C was performed twice. Three colonies were selected from the plates, suspended in 1 mL of PBS, plated on LB plates containing 10% (wt/vol) sucrose, and incubated at 30°C overnight (double crossover). The recombinants were confirmed to be deficient in the target gene using Sanger sequencing (Biotechnology Division, Department of Biotechnology, Tokyo Institute of Technology, Japan). The above method was performed sequentially to delete multiple genes from a single strain, using the corresponding plasmid for each target gene.

The plasmid pTV118N (Takara, Japan) was employed for the expression of the ferrichrome-iron receptor (FhuA) protein. The sequences of both the *fhuA* gene and the plasmid were amplified and combined using the NEBuilder Hifi DNA assembly (New England Biolabs, USA). The assembled plasmid was introduced into *E. coli* JM109 using the heat shock method and cultured on an ampicillin LB plate.

## RESULTS

### Phage isolation and characterization

A novel bacteriophage, SP15, was isolated from a wastewater treatment plant in Tokyo, using *E. coli* O157 as a propagation host. We tested the host range of SP15 and compared it with that of our previously isolated phage PP01, a Myovirus phage belonging to *Tequatrovirus* (34). Unlike PP01, which specifically infects O157, SP15 exhibited a broad host range and infected various strains of *E. coli* (Fig. 1A). Morphological and genomic analyses and several physiological tests were performed to identify and characterize SP15. This bacteriophage belonged to the Siphovirus group based on the morphology observed, featuring a capsid head connected to a long noncontractile tail (Fig. 1B). The latent period and burst size of the phage were 20 min and 138 PFU/cell, respectively (Fig. 1C).

Whole-genome sequencing revealed that the SP15 genome consisted of 110,964 bp with 39% G + C content, 163 open reading frames, and 23 tRNAs. The phage could be classified as a T5-like coliphage (Fig. 1D) under *Tequintavirus*, with 90.45% genome identity to T5 (genome accession number: AY543070). Owing to the absence of sequences encoding integrase, recombinase, repressors, and excisionase in its genome, the SP15 phage can be considered a lytic virus. Furthermore, SP15 had no virulence factors or antibiotic resistance genes in its genome, confirming that it is a genetically safe phage suitable for phage therapy.

**Fig 2:**
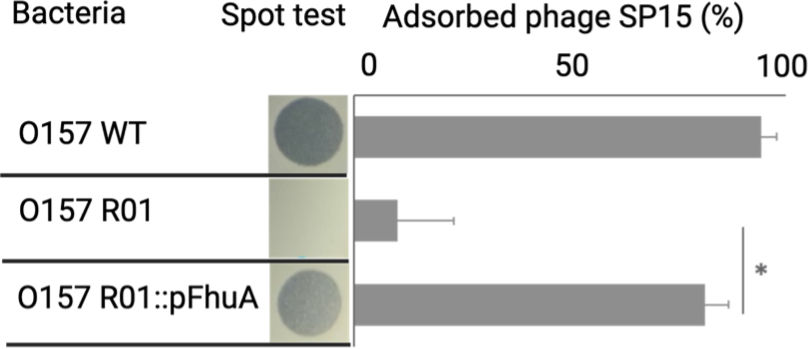
Receptor identification of SP15 using spontaneous mutant bacteria with truncated FhuA. An adsorption assay was performed on the wild-type strain (O157 WT), the spontaneous mutant SP15-resistant O157 harboring truncated FhuA^Trp511Ter^ (O157 R01), and the SP15-resistant O157 complemented with FhuA in *trans* (O157 R01::FhuA). Statistical significance (*P* < 0.01) was indicated by an asterisk.

The adsorption of phages to bacterial receptors is the initial crucial step in phage infection. To identify the receptor of the SP15 phage, it was co-cultivated with O157, and strains that showed resistance to SP15 phage infection were selected. Sequencing of these strains revealed a mutation in which the 511th tryptophan in the *fhuA* was altered to a stop codon (Table S3). Complementation of *fhuA* in this mutant strain with a plasmid restored the adsorption of the SP15 phage, suggesting that FhuA is the receptor for the SP15 phage (Fig. 2).

### Combination of the SP15 phage and fosfomycin suppressed the development of resistance

**Fig 3:**
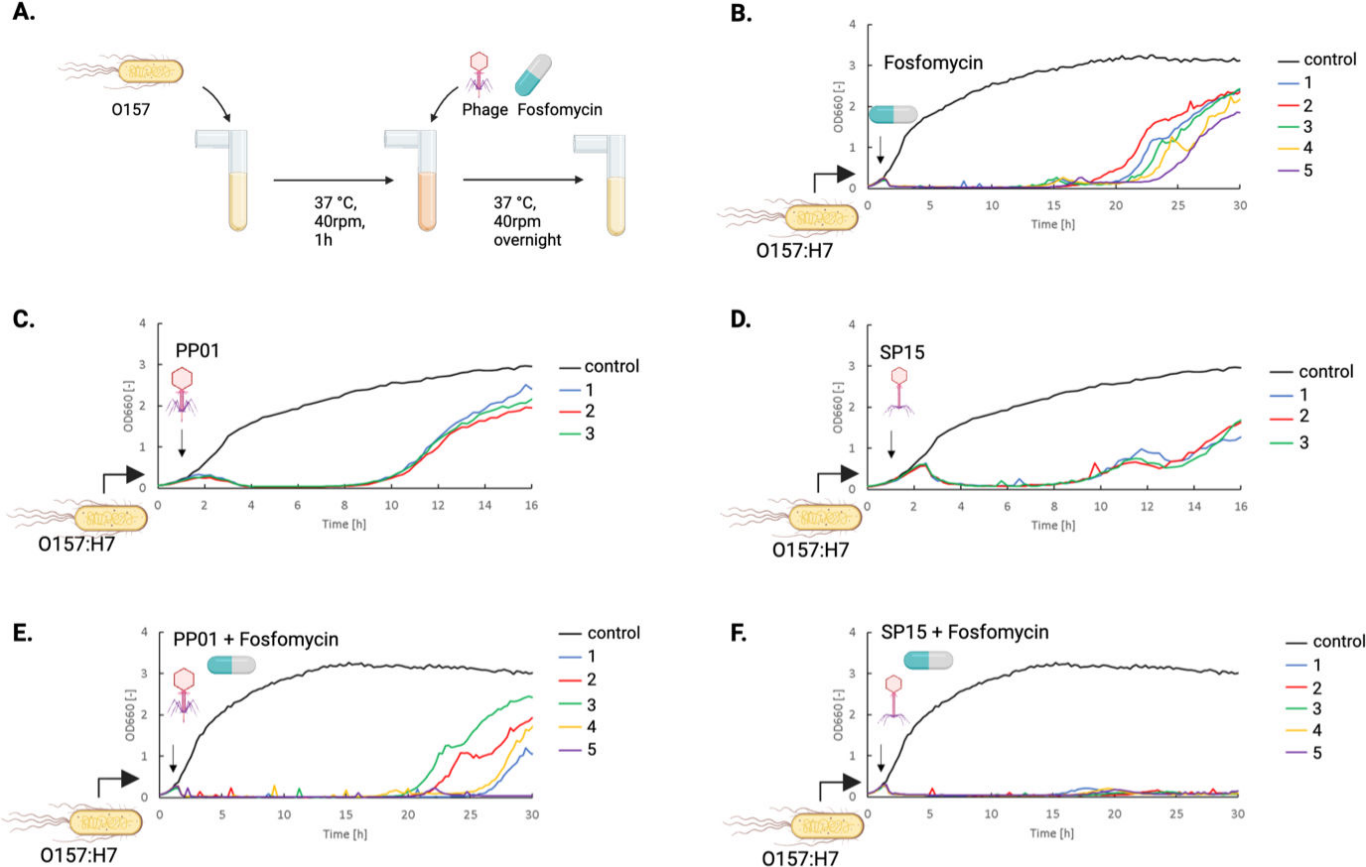
Phage and antibiotic combinations reduce the emergence of resistant strains of O157 more than either treatment alone. (**A**) Illustration of co-culture of phage and bacteria with or without adding fosfomycin. (**B**) Bacterial lysis curve under fosfomycin treatment. Fosfomycin with a final concentration of 4 µg/mL was added into the culture 1 h after bacterial addition. The same experiment was performed in five different runs. (**C**) Bacterial lysis curve under phage PP01 treatment. The same experiment was performed in three different runs. (**D**) Bacterial lysis curve under phage SP15 treatment. The same experiment was performed in three different runs. (**E**) Bacterial lysis under fosfomycin and phage PP01 treatment. The same experiment was performed in five different runs. (**F**) Bacterial lysis under fosfomycin and SP15 treatment. The same experiment was performed in five different runs. The phage at an MOI = 1 or fosfomycin at a final concentration of 4 µg/mL was added 1 h after bacterial addition.

To evaluate the synergistic effects of phages and antibiotics, we examined the effectiveness of bacteriophages SP15 and PP01, which infect O157, along with fosfomycin, a commonly used antibiotic against O157, in killing bacteria (Fig. 3A). Treatment with fosfomycin alone effectively suppressed the growth of O157 for approximately 20 h (Fig. 3B), whereas treatment with either PP01 or SP15 was effective for less than 10 h (Fig. 3C and D). When either PP01 or SP15 was added 1 h after the start of the bacterial culture, the absorbance of the culture medium approached zero 3 h later (Fig. 3C and D). However, an increase in turbidity was observed at around 8 h, likely due to the emergence of phage-resistant bacteria. Both the PP01 and SP15 phages exhibited similar bactericidal curves against O157, but when combined with fosfomycin, different bactericidal curves were observed, depending on the phage type. In the combination of PP01 and fosfomycin, an increase in turbidity was observed in four out of five experiments (Fig. 3E). However, in the combination of SP15 and fosfomycin, no appearance of resistant bacteria was observed in any of the experiments for at least 30 h (Fig. 3F).

### Combination therapy delayed the occurrence of fosfomycin-resistant O157

**Fig 4:**
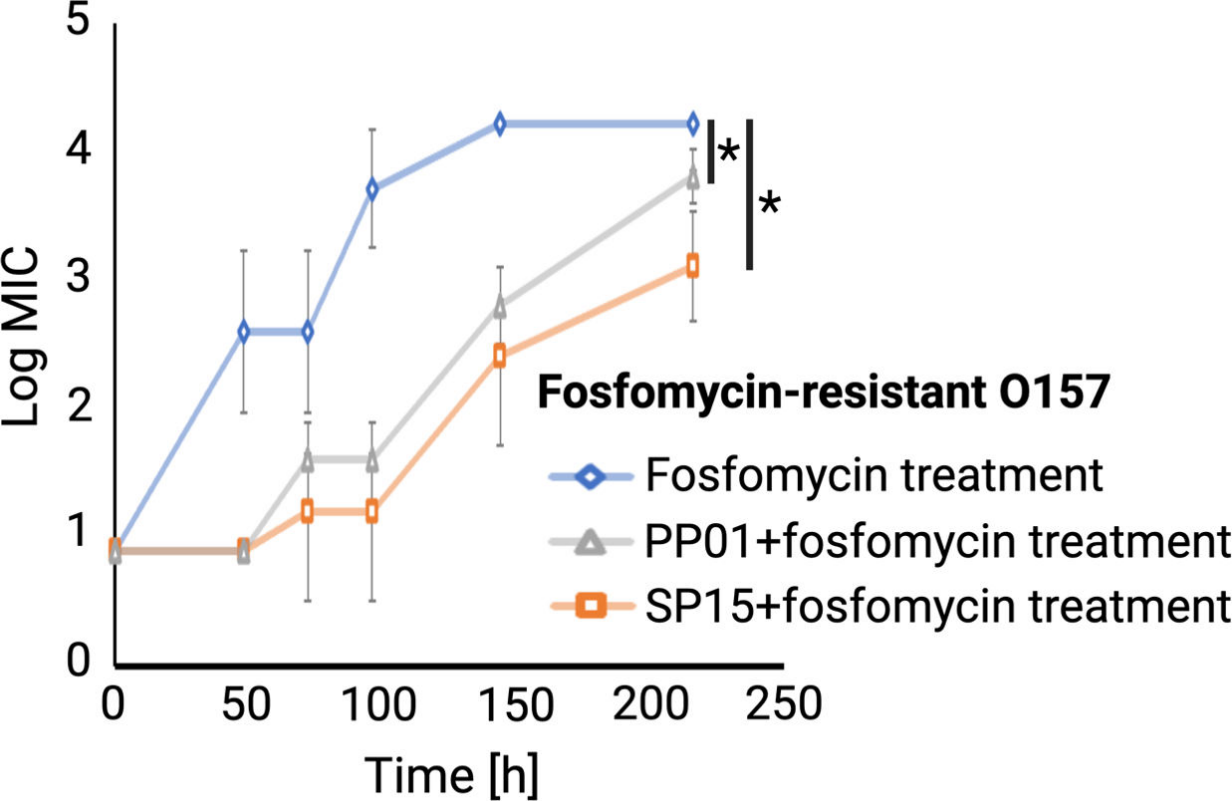
The MIC value of fosfomycin of bacteria isolated from each round of fosfomycin treatment (blue diamond), co-culture with PP01 and fosfomycin (gray triangle), and co-culture with SP15 and fosfomycin (orange square). Statistical difference (*P* < 0.05) is indicated by an asterisk.

Next, to investigate whether combination therapy suppresses the emergence of antibiotic resistance, the MIC values of O157 cultured in the presence of fosfomycin, PP01 + fosfomycin, and SP15 + fosfomycin were measured. We found that the MIC of fosfomycin against wild-type O157 (ATCC 43888) was 16 µg/mL. According to the criteria for fosfomycin resistance established by the Clinical and Laboratory Standards Institute, bacteria with MIC values of ≥256, 128, and ≤64 µg/mL were considered resistant, intermediate resistant, and sensitive bacteria, respectively. Therefore, the wild-type O157 strain used in this study, with an MIC of 16 µg/mL, is considered sensitive to fosfomycin. In the combined treatment of fosfomycin and phages (either PP01 or SP15), the emergence of fosfomycin-resistant O157 strains was significantly delayed (Fig. 4). When treated with both phages and fosfomycin, the MIC for fosfomycin in O157 was approximately 2.5 orders of magnitude lower than when treated with fosfomycin alone.

### Gene mutations acquired in O157 co-cultured with phage and fosfomycin

O157 strains that acquired phage resistance through the co-culture of phages and bacteria in the presence or absence of fosfomycin were subjected to whole-genome sequencing. Comparisons between the resistant strains and the wild-type O157 revealed mutations that confer resistance to phages and fosfomycin (Table S3). The number of mutations acquired in fosfomycin-resistant O157 was the highest, with 9 insertion/deletion (indel) mutations and 35 point mutations. In contrast, PP01-resistant or SP15-resistant O157 exhibited only two point mutations each. The SP15 + fosfomycin-resistant strain exhibited four point mutations and five indel mutations, while the PP01 + fosfomycin-resistant strain displayed five point mutations and two indel mutations. In PP01-resistant O157, a nonsense mutation was identified in which the 76th glutamine of outer membrane protein C (*ompC*) (locus tag: FNZ21_13245), the receptor of PP01 (34, 35), was replaced by a stop codon. In addition, arginine at position 143 of the glycosyltransferase (FNZ21_14180), an enzyme involved in the biosynthesis of oligosaccharides and polysaccharides, was replaced with a stop codon. In SP15-resistant O157, a nonsense mutation was identified in which a stop codon replaced tryptophan at position 511 of *fhuA* (FNZ21_00755). In addition, the proline at position 696 of the DEAD/DEAH box helicase (FNZ21_02015), which is involved in various aspects of RNA metabolism, was replaced by leucine. In fosfomycin-resistant O157, mutations were found in two transporters: hexose phosphate transporter (*uhpT*) (FNZ21_06865) and glycerol-3-phosphate transporter (*glpT*) (FNZ21_13145). A single base deletion of glycine at position 141 in *uhpT* caused a frameshift, and the amino acid at position 202 was replaced with a stop codon. In addition, the glycine at position 358 was replaced by serine in *glpT*. In PP01 + fosfomycin-resistant O157, mutations in *uhpT* and *glpT* were also observed: a stop codon in *uhpT* replaced serine at position 5, and 555 bp from the stop codon was deleted in *glpT*. In contrast, no mutations were found in *ompC*, the PP01 receptor. In SP15 + fosfomycin-resistant O157, there were no mutations in *uhpT*, but there was a 57-bp deletion within the gene encoding *uhpA* (FNZ21_06880), the activator of UhpT (44, 45). In addition, the aspartic acid at position 88 of *glpT* was replaced with glutamic acid. Moreover, there were two mutations in *fhuA*, with a substitution of aspartic acid at position 218 for asparagine and a 69-bp deletion.

### Identification of genetic determinants responsible for the resistance phenotype against phage and fosfomycin

**Fig 5:**
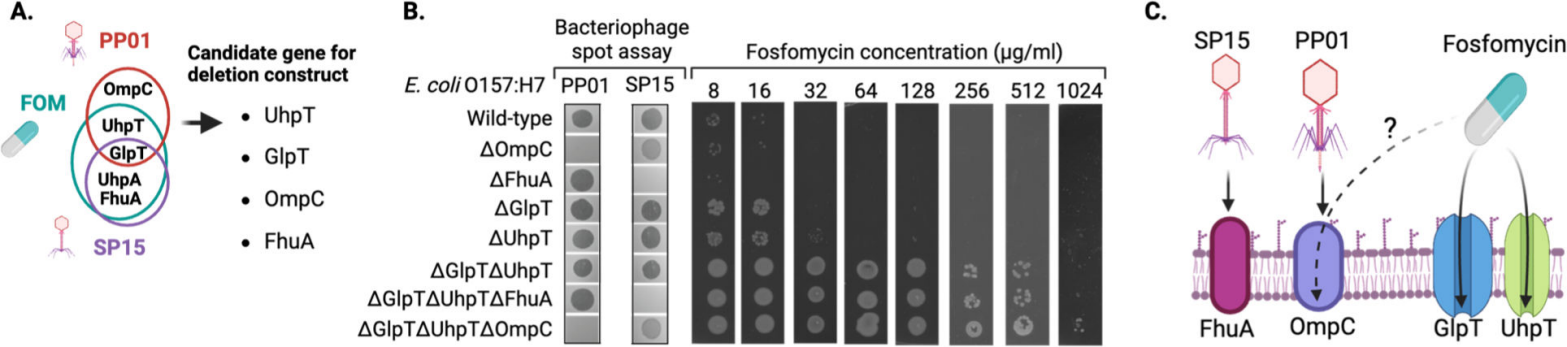
Identification of a prevalent mutation in antibiotic-resistant bacteria following fosfomycin treatment, phage treatment alone, or combined phage and fosfomycin therapy. (**A**) Detection of five commonly mutated genes in the context of phage and antibiotic treatment. Given the dependence of *uhpT* promoter activity on *uhpA*, a deletion mutant was constructed for four genes (*uhpT*, *glpT*, *ompC*, and *fhuA*). (**B**) Alterations in the sensitivity to the phage and changes in the MIC values of fosfomycin observed in the deletion mutant. (**C**) Schematic illustration depicting the phage receptor and fosfomycin uptake channel, as observed in this study.

To determine whether mutations acquired through co-culture experiments with fosfomycin or phages were responsible for fosfomycin and phage resistance in O157, various O157 deletion mutants were generated (Fig. 5A). Candidate genes for deletion included *ompC*, which exhibited mutations with PP01 treatment; *uhpT*, which had common mutations with fosfomycin and PP01 treatment; *uhpA* and *fhuA*, which had common mutations with fosfomycin and SP15 treatment; and *glpT*, which had mutations common to fosfomycin, PP01, and SP15 treatments. UhpA is already known as the activator of UhpT (44, 45). The observed trait associated with *uhpA* mutations (resistance to fosfomycin) was presumed to result from inactivation of UhpT. Therefore, UhpA was excluded from the list of candidate genes for deletion. We confirmed that PP01 and SP15 could not form plaques in the *ompC* and *fhuA* deletion mutants, respectively, suggesting that ompC is a receptor of PP01, whereas FhuA is a receptor of SP15 (Fig. 5B). This result is consistent with previous reports indicating that OmpC acts as the PP01 receptor (34, 35). Furthermore, SP15 did not adhere to the O157 *fhuA* deletion mutant, but adherence was restored upon complementation with a plasmid-expressing FhuA, confirming that FhuA functions as the receptor for SP15 (Fig. 2). Single-deletion mutants lacking the phage receptors OmpC or FhuA showed no change in fosfomycin MIC values. The fosfomycin MIC values for the individual deletion strains of *uhpT* and *glpT* were 32 and 16 µg/mL, respectively. With the wild-type O157 MIC at 16 µg/mL, an increase in MIC was observed only in the *uhpT* deletion strain. The double-deletion strain lacking both *uhpT* and *glpT* (Δ*uhpT*Δ*glpT*) showed no change in sensitivity to phages compared to the wild type. However, the Δ*uhpT*Δ*glpT* strain exhibited a significant increase in fosfomycin MIC for O157 (≥256 µg/mL), leading to high-level fosfomycin resistance. These results indicate that UhpT and GlpT act as fosfomycin transporters. Interestingly, the triple-deletion mutant lacking *ompC*, *uhpT*, and *glpT* (Δ*uhpT*Δ*glpT*Δ*ompC*) showed a twofold increase in fosfomycin MIC compared to the Δ*uhpT*Δ*glpT* mutant. These results suggest the potential involvement of OmpC in the bacterial uptake of fosfomycin.

## DISCUSSION

The combination of phages and antibiotics has been widely used to enhance the eradication of drug-resistant bacteria and mitigate the spread of antibiotic resistance worldwide (8, 46, 47). Several studies have examined the mechanisms underlying phage-antibiotic synergy. Owing to the selection pressure on bacteria caused by phage infection, some toxicity, drug sensitivity, and growth factors are lost, and phage-resistant strains are often less toxic, more sensitive to antibiotics, and grow slower than wild-type strains (46, 47). Our study showed that combining phages with antibiotics can reduce resistant clones, but the type of phage utilized will affect the outcome (Fig. 3). The combination of fosfomycin and SP15 showed the best inhibition of resistant clone development.

Phage adsorption is the first critical step for phages to infect bacterial hosts; therefore, receptor mutations are commonly found in bacteria as a potent defense strategy to escape phage predation (12, 48–51). Although fosfomycin has been reported to be effective in eradicating O157 (28), the rapid emergence of fosfomycin-resistant O157, which has an MIC 1,024 times higher than that of the O157 wild type, indicates that O157 can easily develop resistance against fosfomycin. Whole-genome analysis of fosfomycin-resistant O157 revealed mutations in the genes encoding the two transporters (UhpT and GlpT) that were previously reported to take up fosfomycin (44, 45). In addition, numerous genetic mutations were identified in fosfomycin-resistant clones. We believe that the accumulation of these mutations leads to a decrease in fosfomycin permeability, contributing to an increase in resistance levels (Table S3). However, the mechanism behind these changes remains unknown.

When a combination of phages and antibiotics was added to O157, the emergence of fosfomycin-resistant bacteria was more effectively inhibited than when phages were used alone. In particular, the combination of SP15 and fosfomycin significantly inhibited the emergence of resistant bacteria compared to the combination of PP01 and fosfomycin. This suggests that an optimal combination of phage and antibiotic therapy may exist in antimicrobial treatment. Throughout the five rounds of co-culture, the average MIC values of resistant O157 from PP01 + fosfomycin and SP15 + fosfomycin were significantly lower than those of resistant O157 from fosfomycin treatment alone (Fig. 3), suggesting that the combined use of phage and antibiotics reduced the level of antibiotic resistance.

The O157 strains treated with PP01 + fosfomycin exhibited mutations in the fosfomycin transporters (*uhpT* and *glpT*). No mutations were observed in the identified *ompC* gene, which serves as the receptor for the PP01 phage in the same O157 strain. However, a point mutation was identified in the *hldE* gene (FNZ21_08945), which is associated with lipopolysaccharide synthesis. The mutation in *hldE* might have hindered PP01 adsorption to the host, possibly due to the two-step adsorption process of PP01 to O157. The first step involves the reversible attachment of the long tail fiber (gp38) to the host receptor (OmpC), followed by the irreversible binding of the short tail fiber (gp12) to lipopolysaccharide (34, 35, 38). In O157 obtained through treatment with SP15 + fosfomycin, mutations were observed in two transporters, *uhpT* and *glpT*, which are responsible for fosfomycin uptake, as well as in *fhuA*, the receptor for SP15. These results suggest that O157 can simultaneously acquire mutations in the phage receptor and the fosfomycin uptake channel to escape suppression by phage and fosfomycin.

Deleting the phage receptor gene resulted in the loss of the plaque-forming ability of each phage. This is thought to be due to the inability of phage ligands to attach to the host receptors (Fig. 2 and Fig. 5B). Strains lacking one transporter (UhpT or GlpT) did not show a significant increase in the MIC of fosfomycin compared to the wild type; however, when both were deleted, the MIC was significantly increased, indicating that fosfomycin could enter the cell when one of the transporters was available. Cases of fosfomycin resistance owing to reduced permeability caused by mutations in UhpT and GlpT transporters have been reported in a previous study (28). In the current study, we generated a strain deficient in different genes for the phage receptor and fosfomycin uptake channel (Fig. 5B). The phage sensitivity of *ΔuhpTΔglpTΔompC* was comparable to that of *ΔompC*, but the MIC values of fosfomycin in *ΔompC* and *ΔuhpTΔglpT* were 16 and 512 µg/mL, respectively, whereas that of *ΔuhpTΔglpTΔompC* was 1,024 µg/mL. The absence of uhpT and glpT transporters likely contributes to a substantial reduction in fosfomycin permeability into bacterial cells, leading to the observed increase in resistance. As the MIC value for fosfomycin in Δ*uhpT*Δ*glpT*Δ*ompC* increased compared to the MIC value for fosfomycin in Δ*uhpT*Δ*glpT*, OmpC may also be involved in the permeability of fosfomycin. Thus, it is suggested that the *ompC* mutation acquired for phage resistance may also increase fosfomycin resistance. Our study highlighted the potential use of phage and antibiotic treatment to control O157; however, we should be more selective in determining which type of phage should achieve the best outcome.

Phage-resistant bacteria typically lack surface components that are often involved in pathogenicity (49). Therefore, even if phages fail to eradicate these bacteria, they can still reduce the pathogenicity of the bacteria. Our study also showed that O157 that resists the phage alone or the combination of phage and antibiotic has evolved mutations in genes encoding membrane proteins and lipopolysaccharide (Table S3), both of which have been reported to be virulence factors in gram-negative bacteria (49, 52–58). However, our study was limited to *in vitro* analysis. Additional *in vivo* experiments are crucial for a more comprehensive understanding of the potential use of combined phage-antibiotic therapy before clinical application. Furthermore, while our current study is limited to a single-phage application, we postulated that combining numerous phages as a cocktail with antibiotics may considerably decrease resistant bacteria and boost their therapeutic efficacy.

In this study, the bactericidal effects and resistance development of antibiotic and bacteriophage monotherapy were compared with combination therapy targeting O157. However, phage therapy presents challenges in clinical application, including difficulties in standardization and safety concerns associated with unidentified phage genes. Furthermore, the complex pharmacokinetics of phages hinder the determination of optimal dosing regimens. Despite these challenges, recent advancements in phage therapy have shown promising results (8, 10, 11, 59). Building on the findings of this study, future research is anticipated to explore the effectiveness and safety of simultaneous administration of antibiotics and phages.

In conclusion, we found that the phages used in this study exploited different receptors: PP01 utilized OmpC, whereas SP15 recognized FhuA (Fig. 5C). Deletion of either *glpT* or *uhpT* alone did not cause a significant change in fosfomycin MIC values (Fig. 5B). However, when both transporters were deleted, there was a substantial increase in the MIC values. This suggests that fosfomycin can enter bacteria through at least two different receptors: GlpT and UhpT. In strains lacking both fosfomycin channels, further deletion of *ompC* resulted in an additional increase in MIC values. This suggests that fosfomycin may also utilize OmpC to enter bacterial cells.

## Data Availability

The complete genomic sequence data of the wild-type *Escherichia coli* O157 (strain ATCC 43888) are accessible through the National Center for Biotechnology Information (NCBI) with accession number CP041623. The wild-type phages PP01 and SP15 are also accessible at the DNA Data Bank of Japan (DDBJ) under accession numbers LC348379 and AP019559, respectively.

## SUPPLEMENTAL MATERIAL

Supplemental material spectrum.00427-23-s0001.docx Tables S1-S3.

ASM does not own the copyrights to Supplemental Material that may be linked to, or accessed through, an article. The authors have granted ASM a non-exclusive, world-wide license to publish the Supplemental Material files. Please contact the corresponding author directly for reuse.
