# Supplementary material for: Selective bacteriophages reduce the emergence of resistant bacteria in bacteriophage-antibiotic combination therapy
Source: Microbiol Spectr. 2024 May 2;12(6):e00427-23. doi: 10.1128/spectrum.00427-23 (PMC11237537; doi:10.1128/spectrum.00427-23)
Supplement: Supplemental material — Tables S1-S3. [file spectrum.00427-23-s0001.docx]

Supplementary Table S1.

Bacteria, Bacteriophage, and plasmid used in this study

| **Strain name** | **Description** | **Source** |
| --- | --- | --- |
| **Bacteria** |  |  |
| *E. coli* O157:H7 ATCC43888 | Wild type (no shiga toxins 1 and 2) | This study |
| R5-PP01 | PP01-resistant O157:H7 (Round 5th) | This study |
| R5-SP15 | SP015-resistant O157:H7 (Round 5th) | This study |
| R5-FOM | Fosfomycin-resistant O157:H7 (Round 5th) | This study |
| R5-PF | PP01+FOM-resistant O157:H7 (Round 5th) | This study |
| R5-SF | SP015+FOM-resistant O157:H7 (Round 5th) | This study |
| *E. coli* O157:H7 EDL933 |  | From previous study (1) |
| *E. coli* O157:H7 CR-3 |  | From previous study (1) |
| *E. coli* O116:H10 ATCC23541 |  | From previous study (1) |
| *E. coli* O157:H19 A2 |  | From previous study (1) |
| *E. coli* O157:H37 CE273 |  | From previous study (1) |
| *E. coli* K12 JM109 |  | Takara, Tokyo, Japan |
| *E. coli* K12 JM110 |  | Jichi Medical Univ collection |
| *E. coli* K12 W3110 |  | Jichi Medical Univ collection |
| *E. coli* C |  | Jichi Medical Univ collection |
| *E. coli* C600 |  | Jichi Medical Univ collection |
| *E. coli* BL21 |  | Takara, Tokyo, Japan |
| *E. coli* DH10B |  | Takara, Tokyo, Japan |
| *E. coli* DH5α |  | Takara, Tokyo, Japan |
| *E. coli* MC101 |  | Jichi Medical Univ collection |
| *E. coli* Top10 |  | Jichi Medical Univ collection |
| *E. coli* HST08 |  | Takara, Tokyo, Japan |
| *E. coli* O157:H7 ΔuhpT | *uhpT* knockout O157 | This study |
| *E. coli* O157:H7 ΔglpT | *glpT* knockout O157 | This study |
| *E. coli* O157:H7 ΔuhpTΔglpT | *uhpT* and *glpT* O157 | This study |
| *E. coli* O157:H7 ΔompC | *ompC* knockout O157 | This study |
| *E. coli* O157:H7 ΔfhuA | *fhuA* knockout O157 | This study |
| *E. coli* O157:H7 ΔuhpTΔglpTΔompC | *uhpT*, *glpT* and *ompC* O157 | This study |
| *E. coli* O157:H7 ΔuhpTΔglpTΔfhuA | *uhpT*, *glpT* and *fhuA* O157 | This study |
|  |  |  |
| **Phage** |  |  |
| PP01 | Wild type, infectious to O157:H7 | Previous study (1) |
| SP15 | Wild type, infectious to O157:H7 | This study |
|  |  |  |
| **Plasmid** |  |  |
| pKOV | Temperature-sensitive vector; Cmr, *sacB* | From George Church (Addgene plasmid # 25769; http://n2t.net/addgene:25769 ; RRID:Addgene_25769) |
| pTV118N | Amp^r^, *lac* promoter | Takara, Tokyo, Japan, # 3328 |
| pK-uhpT | For knockout of O157 *uhpT* | This study |
| pK-glpT | For knockout of O157 *glpT* | This study |
| pK-ompC | For knockout of O157 *ompC* | This study |
| pK-fhuA | For knockout of O157 *fhuA* | This study |
| pFhuA | For complementation of *fhuA* | This study |

Supplementary Table S2. Primers used in this study.

| Primer name | Description | Sequence (5’→3’) |
| --- | --- | --- |
| F-up-del-uhpT | Primer to construct pK-uhpT | GCGGGATCCCAGAATGTTCCCACAAAGAG |
| R-up-del-uhpT |  | GGGCAAAAGTCACCAGTTACGAAAGCCAGCATGGGTTACTC |
| F-dw-del-uhpT |  | TCAGGAGTAACCCATGCTGGCTTTCACGTAACTGGTGACTTTTGC |
| R-dw-del-uhpT |  | GCGGTCGACATTGATTACGCACTCCCACTC |
| F-up-del-glpT | Primer to construct pK-glpT | GCGGGATCCCCGCCAATGATAATCACGTC |
| R-up-del-glpT |  | TTTCAGCGTCAATTTCATGCCAAATACTCAACATTGAAAGCCT |
| F-dw-del-glpT |  | ACGGAGGCTTTCAATGTTGAGTATTTGGCATGAAATTGACGCTGA |
| R-dw-del-glpT |  | GCGGTCGACCCTGGATAAGTCTGCACTTT |
| F-up-del-ompC | Primer to construct pK-ompC | GCGGGATCCGAGGCATCCGGTTGAAATAG |
| R-up-del-ompC |  | GCAGGCCCTTTGTTCGATATCAATCTTTAACTTTCATGTTATTAAC |
| F-dw-del-ompC |  | GAGGGTTAATAACATGAAAGTTAAAGATTGATATCGAACAAAGGGC |
| R-dw-del-ompC |  | GCGGTCGACCACGGCGATAAACTTTGCGA |
| F-up-del-fhuA | Primer to construct pK-fhuA | GCGGGATCCGTCAGGCAATCCGTTTGATC |
| R-up-del-fhuA |  | GCCAACTTGTGAAACAGGCACGGGAACGCGCCATTGGTATATC |
| F-dw-del-fhuA |  | CAGAGATATACCAATGGCGCGTTCCCGTGCCTGTTTCACAAGTTG |
| R-dw-del-fhuA |  | GCGGTCGACTAAGCCAACCAGCGAGATAG |
| F-fhuA | Primer to construct pFhuA | CAGGAAACAGACATGGCGCGTTCCAAAACTGCTCAGCCAAAA |
| R-fhuA |  | CCGAGCTCGAATTTTAGAAACGGAAGGTTGCGGTTGCAAC |
| FpTV118 |  | CTTCCGTTTCTAAAATTCGAGCTCGGTACCCGGGGATCCT |
| RpTV118 |  | GGAACGCGCCATGTCTGTTTCCTGTGTGAAATTGTTATCC |

**Supplementary Table. S3**

Mutation identified in resistant bacteria.

| Bacteria | Mutation | | Gene | Product |
| --- | --- | --- | --- | --- |
| PP01-resistant | C→T | Gln76Ter | *ompC_1* | porin OmpC (FNZ21_13245) |
|  | A→T | Arg143Ter | *-* | Glycosyltransferase (FNZ21_14175) |
| SP015-resistant | C→T | Trp511Ter | *fhuA* | ferrichrome porin FhuA (FNZ21_00755) |
|  | G→A | Pro696Leu | *-* | DEAD/DEAH box helicase ( FNZ21_02015) |
| FOM-resistant | G→A | Thr325Ile | *aceE* | pyruvate dehydrogenase (acetyl-transferring), homodimeric type (FNZ21_00935) |
|  | T→C | Trp545Arg | *-* | HTH-type transcriptional regulator SgrR (FNZ21_01150) |
|  | G→A | Ala102Val | *osmY* | molecular chaperone OsmY (FNZ21_01650) |
|  | GCGCCA del | 72Leu 73Ala del | *mutL* | DNA mismatch repair endonuclease MutL (FNZ21_02765) |
|  | T→C | Val174Ala | *iclR* | glyoxylate bypass operon transcriptional repressor IclR (FNZ21_03605) |
|  | G→A | Arg127Cys | *-* | serine/threonine protein kinase (FNZ21_04455) |
|  | T→C | Val215Ala | *malt* | HTH-type transcriptional regulator MalT (FNZ21_05100) |
|  | A→G | Cys66Arg | *-* | hypothetical protein |
|  | 282bp del | | *espF* | type III secretion system LEE effector EspF (FNZ21_06600) |
|  | C del | Gly141fs 202Ter | *uhpT* | hexose-6-phosphate transporter (FNZ21_06865) |
|  | C→T | Trp222Ter | *-* | Cytotoxin (FNZ21_09295) |
|  | C→T | His149Tyr | *lysA* | diaminopimelate decarboxylase (FNZ21_10155) |
|  | T→C | Asn71Asp | *-* | Kinase (FNZ21_10495) |
|  | C→T | Val36Ile | *intS* | prophage integrase IntS (FNZ21_12615) |
|  | C del | Pro99fs 132Ter | *-* | hypothetical protein |
|  | G→T | Glu149Asp | *-* | hypothetical protein |
|  | ins A | Glu20fs 27Ter | *rhmD* | L-rhamnonate dehydratase (FNZ21_13110) |
|  | G→A | Gly358Ser | *glpT* | glycerol-3-phosphate transporter (FNZ21_13145) |
|  | C→T | Gly28Asp | *-* | nickel/cobalt efflux protein RcnA (FNZ21_13830) |
|  | C→T | Glu22Lys | *-* | YjbH domain-containing protein (FNZ21_14960) |
|  | T→C | Gln39Arg | *-* | helix-turn-helix domain-containing protein |
|  | A→G | Lys160Glu | *gap* | type I glyceraldehyde-3-phosphate dehydrogenase (FNZ21_19050) |
|  | A→G | Phe451Leu | *hrpA* | ATP-dependent RNA helicase HrpA (FNZ21_19085) |
|  | T→C | Asn431Ser | *-* | autotransporter outer membrane beta-barrel domain-containing protein (FNZ21_19125) |
|  | G→A | Gly226Asp | *-* | pyruvate kinese I (FNZ21_20965) |
|  | G→A | Ala102Thr | *-* | hypothetical protein |
|  | G→A | Ala283Thr | *pyk* | pyruvate kinese (FNZ21_21890) |
|  | ins T | Asn105fs 116Ter | *-* | leucine-rich repeat domain-containing protein (FNZ21_22470) |
|  | A→C | Thr228Pro | *-* | fimbrial protein (FNZ21_23195) |
|  | ins C | Gly76fs 120Ter | *-* | uracil/xanthine transporter (FNZ21_25615) |
|  | ins C | Asp33fs 48Ter | *sbmA* | peptide antibiotic transporter SdmA (FNZ21_26345) |
| PP01+FOM-resistant | G→C | Val317Cys | *malt* | HTH-type transcriptional regulator MalT (FNZ21_05100) |
|  | A→C | Leu5Ter | *uhpT* | hexose-6-phosphate transporter (FNZ21_06865) |
|  | C→A | Arg396Ser | *hldE* | bifunctional D-glycero-beta-D-manno-heptose-7-phosphate kinase HldE (FNZ21_08945) |
|  | 555bp del | | *glpT* | glycerol-3-phosphate transporter (FNZ21_13145) |
|  | 352bp del | | *glpQ* | glycerophosphodiester phosphodiesterase (FNZ21_13150) |
| SP015+FOM-resistant | C→T | Asp218Asn | *fhuA* | ferrichrome porin FhuA (FNZ21_00755) |
|  | 69bp del | |  |  |
|  | 57bp del | | *uhpA* | transcriptional regulator UhpA (FNZ21_06880) |
|  | G→A | Ser338Phe | *atpA* | F0F1 ATP synthase subunit alpha (FNZ21_07235) |
|  | T→A | Asp88Glu | *glpT* | glycerol-3-phosphate transporter (FNZ21_13145) |
|  | CAATTT del | Phe252 Gln253 del | *-* | Mannose-1-phosphate guanylyltransferase 2 (FNZ21_14220) |
|  | G del | Leu134fs 193Ter | *-* | helix-turn-helix domain-containing protein |
|  | 54bp del |  | *prk* | pyruvate kinase (FNZ21_21890) |
